# Validation of phenomenon and cross-sectional investigation of predictors for a post-COVID-19 surge of osteoporosis outpatients in China

**DOI:** 10.1038/s41598-024-54858-4

**Published:** 2024-02-20

**Authors:** Lei Sun, Yuehua Zhang, Yao Chen, Li Chen, Mei Lei

**Affiliations:** 1 Department of Osteoporosis, Non-Communicable Diseases Research Center, West China-PUMC C.C. Chen Institute of Health, West China School of Public Health and West China Fourth Hospital, Sichuan University, Chengdu, 610041 Sichuan People’s Republic of China; 2 Department of Medical Records, West China School of Public Health and West China Fourth Hospital, Sichuan University, Chengdu, 610041 Sichuan People’s Republic of China; 3 Department of Orthopaedics Medical Center, West China School of Public Health and West China Fourth Hospital, Sichuan University, #18 3Rd Section, Renmin Nan Road, Chengdu, 610041 Sichuan People’s Republic of China

**Keywords:** Post-COVID-19, Influencing factor, Internal and external validation, Osteoporosis, Post-lockdown outpatient volume increases, Osteoporosis, Health services

## Abstract

An unexpected surge of osteoporosis outpatients occurred after COVID-19 lockdown was lifted in China. To confirm the observation and identify possible reasons driving patients care seeking behaviors post-pandemic, we compared the outpatient volumes at the osteoporosis clinic in January through May, 2019–2022 and surveyed seven osteoporosis specialists across China to validate the phenomenon before devising an online questionnaire to collect patients’ characters and physical activity levels. Univariate and binary logistic regression analyses were calculated to identify predictors of post-lockdown care-seeking. We received 480 valid responses, including 397 (82.7%) patients having visited the clinic after lockdown and 83 (17.3%) having not. Four significant predictors were identified, including being female, experiencing pain, aggravating symptoms, and heightened anxiety during lockdown (*P* < 0.05). Both groups experienced lower physical activity levels during lockdown, which however was not a significant predictor (*P* = 0.317). The surge in osteoporosis outpatient visits after COVID-19 lockdown suggests vast latent demand for osteoporosis care accumulated during the pandemic. Four significant factors predict post-lockdown outpatient care-seeking, including being female, experiencing pain and aggravating symptoms, and heightened anxiety levels. Though physical activity levels decreased during lockdown, it failed to predict care-seeking. This demonstrates resilience of osteoporosis patients to resume regular care despite disruption and stress the substantial backlog of unmet healthcare needs.

## Introduction

Since its emergence in late 2019, the severe acute respiratory syndrome coronavirus 2 (SARS-CoV-2) has posed unprecedented challenges globally, prompting nations to implement containment measures such as lockdowns and travel restrictions to mitigate its spread^1,2^. China, the initial epicenter, rapidly enacted strict measures, curbing the virus's transmission effectively^3,4^.

The COVID-19 pandemic has strained healthcare systems worldwide, disrupting routine care and management of chronic conditions due to the reallocation of resources towards managing the crisis^5^. This disruption has particularly impacted the care of chronic diseases, leading to delayed diagnoses and postponed treatments, adversely affecting those reliant on continual medical oversight^6–8^.

Osteoporosis, a prevalent chronic metabolic bone disorder characterized by diminished bone density and heightened fracture risk, exemplifies such a condition. It affects a significant portion of the global and Chinese population, particularly older adults and women, necessitating consistent lifestyle management and treatment adherence^9–13^. The pandemic's disruptions have posed additional challenges for these patients.

Following the relaxation of pandemic-related restrictions in China by February 2022, our osteoporosis outpatient clinic experienced an unforeseen increase in patient volume. Contrary to the prevailing expectations based on existing literature, which anticipated a gradual return to pre-pandemic service levels or a slight decrease^14–17^, our clinic observed a significant surge. While some reports have noted considerable increases in specific services such as elective cardiac surgery due to backlog^18,19^, the dramatic rise in osteoporosis care demand post-lockdown has not been reported. This unexpected trend led us to initiate a comprehensive investigation to understand the underlying factors and potential implications for healthcare delivery.

Our study employs a multi-faceted approach to grasp the full scope of this phenomenon. We begin with a descriptive time-series analysis of our clinic's operational data to internally validate the observed increase. To gauge the broader relevance and compare with trends at other institutions, we conducted a qualitative survey across various healthcare providers. Additionally, a quantitative survey among our patients was undertaken to pinpoint the key factors influencing their decision to seek care post-lockdown. This layered methodology aims to provide a holistic view of the surge in osteoporosis outpatient visits, filling a notable gap in the literature and offering insights that could inform future healthcare strategies in the wake of public health crises.

## Materials and methods

### Validation of the phenomenon

Internal validation: To investigate the observed increase in osteoporosis outpatient visits, we conducted a detailed analysis over a three-month period (March–May 2022) within our clinic. For comparative analysis, we extracted outpatient volume data from our hospital’s operational system, encompassing the timeframe from January to May for the past four years (2019–2022). This longitudinal data provided a comprehensive backdrop against which we could measure the recent surge.

External validation: Recognizing the need to establish whether this trend extended beyond our clinic, we pursued external validation. Considering the proprietary nature of outpatient volume statistics in many Chinese hospitals, we opted for a direct survey approach. We carefully selected seven osteoporosis specialists based on their geographic diversity across China: 1 from another local hospital in Chengdu, Sichuan Province (Western China), 2 from tertiary hospitals in Shanghai (Eastern Coast), 2 from South China (Guangzhou and Shenzhen), 1 from Shenyang in Liaoning Province (Northern China), and 1 from Urumqi in Xinjiang (far western region).

The specialists were approached via telephone, where we inquired if they had noted any recent changes in osteoporosis outpatient volumes. For those reporting an uptick, we delved deeper with follow-up questions to ascertain the onset of this trend and to contextualize the increase relative to previous years. The questions were phrased as “Have you observed any changes in your osteoporosis outpatient volume recently?” and follow-up questions “When did you first notice this increase?” and “How does this increase compare with volumes from previous years, in your estimation?”. All specialists were free to decline the survey, who responded voluntarily.

### Development and validation of survey questionnaire

After validating our observations, we designed an online cross-sectional survey to gather demographic information and explore the osteoporosis-related symptoms, mental status, and physical activity of outpatients during the COVID-19 lockdown. Additionally, we sought to understand the reasons behind their initial visit to our osteoporosis clinic post-lockdown.

Following the initial validation of our observations, we constructed an online cross-sectional survey with 45 questions in 5 sections aimed at collecting demographic data, symptoms, mental health, and physical activity levels among osteoporosis outpatients during the COVID-19 lockdown as well as their reasons for the first visit to the osteoporosis clinic after the lockdown was lifted.

The survey sections included: (1) Sociodemographic and Medical Information, including relevant characteristics; (2) Symptoms, featuring multiple-choice questions on osteoporosis-related symptoms and a 5-point scale for symptom severity assessment during lockdown; (3) Mental Status, assessing changes in depression, anxiety, and overall mental well-being; (4) Reasons for the First Post-Lockdown Clinic Visit, inquiring about healthcare service utilization and motivations for seeking care; and (5) Physical Activity During Lockdown, utilizing a modified International Physical Activity Questionnaire (IPAQ) Long Form (Chinese version) to detail patients' activity levels, with adjustments to reflect the lockdown period^20,21^.

Notably, we initially considered employing established tools for depression and anxiety assessments. However, pilot testing indicated that their inclusion would have significantly extended the survey length, potentially reducing response rates and participant engagement. Given our study's broader focus on general mental health trends rather than precise symptomatology, we opted for a concise set of questions to capture overall emotional well-being.

We validated the questionnaire with 50 osteoporosis outpatients, focusing on internal consistency, test–retest reliability, and comparability to the original IPAQ. Cronbach's alpha coefficients ranged from 0.81 to 0.89, indicating high internal consistency. Test–retest reliability was confirmed through consistent responses over a two-week interval, with intraclass correlation coefficients (ICCs) between 0.80 and 0.90. The modified IPAQ's validity was further supported by a Spearman's rank correlation of 0.90 with the original version, affirming its reliability in assessing physical activity levels during the lockdown.

### Cross-sectional survey

We launched the survey using Wenjuanxing (Ranxing Tech, Changsha), an online survey platform widely used by Chinese researchers. A link to the questionnaire was sent to the patients who had visited our osteoporosis clinic previously or recently.

#### Sample size estimation

Admittedly, the initial sample size necessary for our study was calculated using a method more appropriate for prevalence estimation within a population. However, our primary objective was to explore the association and significance of various factors influencing the likelihood of visiting or not visiting an osteoporosis clinic post-COVID-19 lockdown. This methodological issue was highlighted by a peer reviewer, who recommended a more suitable technique described by Tushar Vijay Sakpal^22^.

Acknowledging this insight, we reassessed our sample size using the recommended approach with the following parameters: Primary outcome measure: Visiting or not visiting the osteoporosis clinic after the COVID-19 lockdown. Type I Error (Alpha): 0.05; Type II Error (Beta) and Power (1—Beta): 0.20. Expected proportions in each group: 3:1 (3 for visiting, 1 for not visiting). Hypotheses: Null hypothesis (H0): No difference between groups or no association between variables; Alternative hypothesis (H1): There is a difference or association. Adjustments for multiple testing: Set at 0.05. Sample size distribution: 3:1.

Our reassessment indicated that for a large (0.8), medium (0.5), and small (0.2) effect size, the minimal sample sizes needed were 67, 169, and 1048, respectively. Consequently, our actual sample size (N = 480) was more than adequate to detect large and medium effect sizes, but not small effect sizes. This reassures the validity of our findings for the intended effect sizes, while we acknowledge the limitation in detecting a smaller effect.

### Analyses of influencing factors for seeking outpatient care after COVID-19 lockdown

To discern the factors driving patients to seek outpatient care post-lockdown, we initiated with univariate regression analysis. Subsequently, significant variables from this preliminary analysis were incorporated into a multivariate framework. We constructed a binary logistic regression model using these significant predictors to delve deeper into the impact of each factor. The model's predictive power was evaluated using the area under the receiver operating characteristic (AUC of the ROC) curve.

### Statistical analyses

All statistical analyses were performed using the Statistical Package for the Social Sciences (SPSS) version 26. Descriptive statistics, including means, standard deviations, frequencies, and percentages, were used to summarize the data. The modified IPAQ Long Form scores were calculated according to the original IPAQ's scoring protocol, with physical activity levels categorized as low, moderate, or high based on the established definitions^24^. Both univariate and multivariate logistic regression analyses were undertaken to identify potential predictors influencing the uptick in outpatient visits. *P* < 0.05 was statistically significant.

### Ethical considerations and informed consent

Our study was ethically approved by the Ethics Committee of West China Fourth Hospital, Sichuan University (Approval number HXSYEC2022031) and adhered to the Declaration of Helsinki and relevant regulatory codes. Informed consent was obtained with the survey's introductory statement clarifying that proceeding with the questionnaire implied consent for research use of the responses. Additionally, a confirmation pop-up was presented upon survey completion, reiterating the consent for the utilization of responses in our research.

### Use of large language model

In refining the language of our manuscript, we employed ChatGPT-4.0 (OpenAI, San Francisco, USA) as a tool to enhance the clarity, coherence, and grammatical accuracy of our original writing. The outputs provided by ChatGPT were meticulously reviewed and selectively integrated by our team to ensure that the essence and scientific rigor of our study, including the design, findings, and analytical discourse, remained intact and faithfully represented.

## Results

### Validation of the phenomenon

#### Internal validation

**Figure 1:**
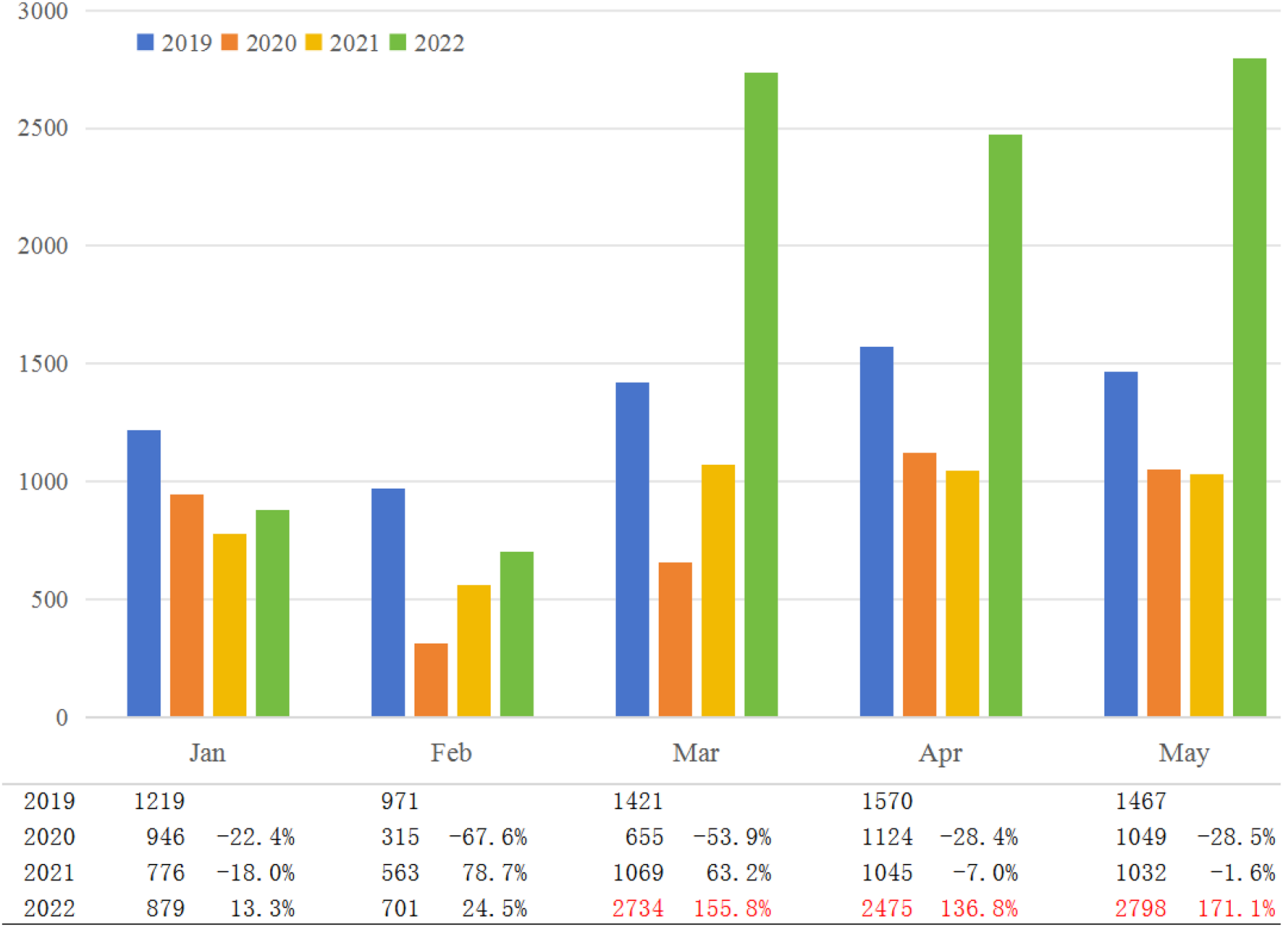
Monthly outpatient volumes at the osteoporosis clinic from January to May, 2019–2022. Monthly outpatient volumes decreased significantly in 2020 after the breakout of the COVID-19 pandemic in December 2019 and gradually recovered in 2021. After the lifting of the lockdown, a sudden surge of osteoporosis outpatients was observed. The table below the figure details the percentage increase in outpatient visits compared to the corresponding months in previous years.

Analysis of outpatient visit data from our osteoporosis clinic for January to May across 2019–2022 revealed distinct patterns (Fig. 1). The 2019 data served as a pre-pandemic baseline, with February traditionally showing the lowest attendance due to the Spring Festival, a period when Chinese families traditionally gather for annual reunions and patients are less likely to seek care. Despite the pandemic's onset in late 2019, this seasonal trend persisted, albeit with reduced overall patient volumes in subsequent years. A notable surge occurred in 2022, post-lockdown, with outpatient numbers more than doubling compared to 2021 and nearly reaching double the pre-pandemic figures of 2019.

#### External validation

Among the seven osteoporosis specialists we consulted across China, five reported significant upticks in outpatient volumes post-lockdown, corroborating our clinic's findings. The other two specialists observed increases, albeit more modest. Given the variability in responses and the inherent limitations of a small specialist sample, these findings underline the necessity for a more extensive quantitative analysis to comprehensively understand the pandemic's long-term effects on osteoporosis outpatient care. Further details on the specialists' demographics and responses are provided in Appendix 1.

### Respondent characteristics

Out of 550 patients approached from January to May 2022, we received 480 valid survey responses (response rate, 87.3%). Among these, 397 (82.7%) had sought care at our clinic post-lockdown, while 83 (17.3%) had not visited post-lockdown. The average age of respondents was 52.3 years, ranging from 19 to 82, with a majority being women (271, 56.5%). The demographic characteristics of the respondents are detailed in Table 1.

**Table 1:** Demographic characteristics of respondents (N = 480).

	*n*	%
Age, yr		
< 30	61	12.7
30–45	122	25.4
45–60	160	33.3
> 60	137	28.5
Gender		
Male	209	43.5
Female	271	56.5
Education level		
Illiterate	10	2.1
Primary school	72	15.0
High school	170	35.4
College or over	228	47.5
Marital status		
Single	51	10.6
Married	381	79.4
Divorced	39	8.1
Widowed	8	1.7
Residency		
Urban	299	62.3
Rural	181	37.7
Living condition		
Alone	51	10.6
With spouse/offspring	429	89.4
Insurance		
None	33	6.9
Urban resident/employee insurance (social, medical insurance)	223	46.5
Rural cooperative medical insurance	137	28.5
Commercial insurance	87	18.1
Time of osteoporosis diagnosis, yr		
< 1	165	34.4
1–3	198	41.3
> 3	117	24.4

Note: yr, year.

### Predictors of osteoporosis outpatient consultation after COVID-19

#### Univariate regression analysis

Univariate analysis identified five key factors significantly associated with the likelihood of seeking osteoporosis care post-lockdown, *i.e.* gender, presence of pain, worsening of symptoms, and anxiety experienced during lockdown (*P* < 0.05). Other demographic and clinical characteristics did not show a significant impact (*P* > 0.05), as detailed in Table 2.

**Table 2:** Predictors of outpatient care seeking among osteoporosis patients after COVID-19 lockdown: Univariate analysis (N = 480).

Characteristic	Patients having not visited clinic (*n* = 83)	Patients having visited clinic (*n* = 397)	X2/t	*P*
Age, yr			0.168	0.983
< 30	10 (12.0%)	52 (13.1%)		
30–45	21 (25.3%)	101 (25.4%)		
45–60	27 (32.5%)	134 (33.8%)		
> 60	25 (30.1%)	110 (27.7%)		
Gender			7.044	0.009
Male	53 (63.9%)	185 (46.6%)		
Female	30 (36.1%)	212 (53.4%)		
BMI	20.190 ± 2.634	20.305 ± 2.549	-0.343	0.732
Education level			4.510	0.211
Illiterate	4 (4.8%)	4 (1.0%)		
Primary school	13 (15.7%)	59 (14.9%)		
High school	29 (34.9%)	141 (35.5%)		
College or over	37 (44.6%)	193 (48.6%)		
Marital status			1.364	0.714
Single	11 (13.3%)	38 (9.6%)		
Married	63 (75.9%)	321 (80.9%)		
Divorced	8 (9.6%)	31 (7.6%)		
Widowed	1 (1.2%)	8 (2.0%)		
Residency			0.010	1.000
Urban	52 (62.7%)	246 (62.0%)		
Rural	31 (37.3%)	152 (38.0%)		
Living condition			0.825	0.364
Alone	11 (13.3%)	38 (9.6%)		
With spouse/offspring	72 (86.7%)	359 (90.4%)		
Insurance			0.245	0.970
None	6 (7.2%)	27 (6.8%)		
Urban resident/employee insurance (social, medical insurance)	40 (48.2%)	181 (45.6%)		
Rural cooperative medical insurance	23 (27.7%)	114 (28.7%)		
Commercial insurance	14 (16.9%)	75 (18.9%)		
Time of osteoporosis, yr			0.948	0.622
< 1	25 (30.1%)	143 (36.0%)		
1–3	36 (43.4%)	160 (40.3%)		
> 3	22 (26.5%)	94 (23.7%)		
Osteoporosis-related symptoms during COVID-19 lockdown				
None	26 (31.3%)	89 (22.4%)	2.431	0.133
Fracture	5 (6%)	23 (5.8%)	0.007	1.000
Pain	10 (12%)	164 (41.3%)	23.036	0.000
Tooth loosening	34 (41%)	151 (38.0%)	0.222	0.637
Reduced body weight	30 (36.1%)	183 (46.1%)	2.421	0.149
Ear ringing	19 (22.9%)	111 (28.0%)	0.760	0.462
Symptom change than before COVID-19 lockdown			33.040	0.000
Much worse	6 (7.2%)	63 (15.9%)		
Somewhat worse	23 (27.7%)	218 (54.9%)		
Not much change	33 (39.8%)	61 (15.4%)		
Somewhat improved	19 (22.9%)	48 (12.1%)		
Much improved	2 (2.4%)	8 (1.8%)		
Experience of anxiety during COVID-19 lockdown			86.634	0.000
Not at all	53 (63.9%)	46 (11.6%)		
A little	22 (26.5%)	177 (44.6%)		
Very much	8 (9.6%)	174 (43.8%)		
Experience of depression during COVID-19 lockdown			0.023	0.989
Not at all	24 (28.9%)	118 (29.7%)		
A little	29 (34.9%)	137 (34.5%)		
Very much	30 (36.1%)	141 (35.8%)		
Mental state change than before COVID-19 lockdown			3.547	0.471
Much worse	3 (3.6%)	12 (3.0%)		
Somewhat worse	16 (19.3%)	71 (17.9%)		
Not much change	28 (33.7%)	116 (29.2%)		
Somewhat improved	32 (38.6%)	191 (47.9%)		
Much improved	4 (4.8%)	8 (2.0%)		
Use of osteoporosis-related medical services during COVID-19 lockdown				
None	32 (38.6%)	151 (38.0%)	0.008	1.000
Online consultation	27 (32.5%)	132 (33.2%)	0.011	0.916
Medication home delivery	29 (34.9%)	143 (36.0%)	0.032	0.893
In-person outpatient consultation	20 (24.1%)	94 (23.7%)	0.010	0.922
Hospitalization	15 (18.1%)	76 (19.1%)	0.052	0.820
Home visit care	0 (0%)	0 (0%)	–	–
Physical activity during COVID-19 lockdown			0.801	0.317
Low	26 (31.3%)	182 (45.8%)		
Moderate	52 (62.7%)	168 (42.3%)		
High	5 (6.0%)	47 (11.8%)		

Note: yr, year.

#### Binary logistic regression analysis

The binary logistic regression model, incorporating significant predictors from the univariate analysis, revealed all four factors as significant determinants of post-lockdown outpatient care-seeking behavior. The model indicated that women were 2.205 times more likely to seek care than men (*P* = 0.020). Patients experiencing pain were 3.919 times more likely to visit the clinic compared to those without pain (*P* = 0.001). A decrease in symptom severity reduced the likelihood of seeking care to 0.543 times the original probability (*P* = 0.000). An increase in anxiety levels was associated with a 5.518-fold increase in the likelihood of seeking care (*P* = 0.000), as shown in Table 3.

**Table 3:** Predictors of outpatient care seeking among osteoporosis patients after COVID-19 lockdown: binary logistic regression modeling (N = 480).

Predictor	B	*P*	*OR*	95% CI
Gender (female)	0.791	0.020	2.205	(1.131, 4.300)
Osteoporosis-related symptoms during COVID-19 lockdown (pain)	1.366	0.001	3.919	(1.692, 9.076)
Symptom change than before COVID-19 lockdown	− 0.610	0.000	0.543	(0.388, 0.760)
Experience of anxiety during COVID-19 lockdown	1.708	0.000	5.518	(3.393, 8.973)
Constant	− 1.806	0.079	0.164	

*OR*, odds ratio; 95% CI 95% confidence interval.

**Figure 2:**
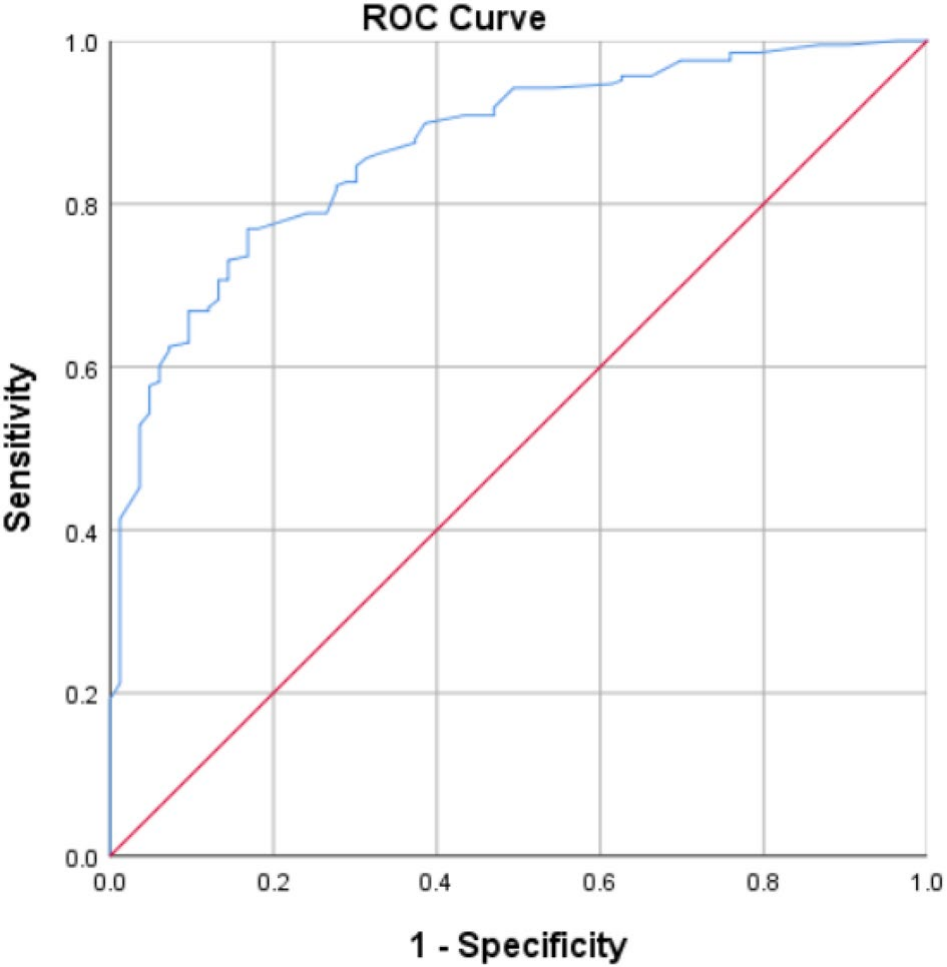
AUC of ROC curve of binary logistic regression modeling of predictors for osteoporosis outpatient care seeking after COVID-19 lockdown.

The predictive accuracy of the model was high, with an AUC of the ROC curve at 0.869 (95% CI 0.826–0.911, *P* < 0.05), indicating the model's strong capability to differentiate between those who would seek care post-lockdown and those who would not (Fig. 2).

### Reasons for the first visit after COVID-19 lockdown lifted

The predominant reason cited by respondents for their initial post-lockdown visit to our osteoporosis clinic was the worsening of symptoms (65.7%), highlighting the immediate healthcare needs that arose due to delayed care during lockdown. Other significant reasons included the prolonged absence of follow-up consultations (48.1%), the need for medication prescriptions (47.6%), anxiety related to their condition (42.6%), the requirement for rehabilitative therapy (42.1%), and scheduled follow-up visits (40.6%). (Table 4).

**Table 4:** Reasons for the first outpatient visit of respondents after COVID-19 lockdown (N = 397).

Reason for first outpatient visit	*n*	%
Initial consultation	78	19.6
Followup visit	161	40.6
Medication prescription	189	47.6
Testing and examination	84	21.2
Rehabilitative therapy	167	42.1
Without followup consultation for too long	191	48.1
Bad or aggravating symptom(s)	261	65.7
Feeling anxious	169	42.6
Updating care plan	54	13.6
Others	37	9.3

### Physical activity during COVID-19 lockdown

Analysis of physical activity levels, as reported through the modified IPAQ, revealed no significant difference between respondents who sought outpatient care post-lockdown and those who did not (*P* = 0.317). The majority of both groups exhibited moderate to low levels of physical activity during the lockdown (some 90% in both groups), with a notable proportion reporting a substantial decrease compared to pre-pandemic levels. (Table 5).

**Table 5:** Physical activity levels of osteoporosis patients during COVID-19 lockdown (N = 480).

	Patients having not visited clinic (*n* = 83)	Patients having visited clinic (*n* = 397)
Physical activity during COVID-19 lockdown		
Low	26 (31.3%)	182 (45.8%)
Moderate	52 (62.7%)	168 (42.3%)
High	5 (6.0%)	47 (11.8%)
Change in physical activity level than before COVID-19 lockdown		
Much lower	42 (50.6%)	221 (55.7%)
Somewhat lower	21 (25.3%)	89 (22.4%)
Not much change	11 (13.3%)	57 (14.4%)
Somewhat higher	4 (4.8%)	23 (5.8%)
Much higher	5 (6.0%)	7 (1.8%)

## Discussion

Our study unveils a post-COVID-19 surge in osteoporosis outpatient visits, a phenomenon previously unanticipated and undocumented. The marked increase in demand for osteoporosis care, following the relaxation of lockdown measures, signals a substantial backlog of healthcare needs that accumulated during the pandemic. This revelation is critical for healthcare providers and policymakers, indicating the need for strategic planning to address the pent-up demand for chronic disease management in the post-pandemic era. It also reflects the resilience and determination of patients to seek continued care for chronic conditions, underscoring the importance of maintaining accessible healthcare services even amidst significant disruptions.

The validation process, employing both internal and external analyses, confirmed the widespread nature of this surge across various regions in China, establishing a solid foundation for our subsequent analyses. Notably, our study identified gender, particularly being female, as a significant predictor for post-lockdown healthcare-seeking behavior. This aligns with broader observations in healthcare, where women are generally more likely to seek medical attention^23,24^. The higher incidence of osteoporosis among women, especially post-menopausal, may further amplify this trend, emphasizing the need for gender-sensitive healthcare strategies^25,26^.

The impact of the pandemic on mental health, notably increased stress and anxiety levels, also emerged as a significant driver for seeking care, potentially exacerbating existing health conditions or spurring the need for medical attention. Our findings are consistent with existing literature on aggravated gravity of depression and anxiety among diseased and general populations^27–29^. This aspect highlights the intersection between mental and physical health, suggesting that healthcare systems should integrate psychological support within chronic disease management frameworks, particularly in the wake of public health crises.

The pandemic-induced stress and changes in daily routines may have disproportionately affected women, potentially driving them to seek healthcare more promptly post-lockdown^30,31^. Research indicates that women report higher stress and anxiety levels, a trend that persisted during the COVID-19 crisis^32^. Healthcare systems should consider to implement gender-sensitive strategies in care delivery during public health emergencies.

The observation that pain significantly influenced outpatient care-seeking during the lockdown aligns with the basic health-seeking behavior principle: noticeable symptoms frequently prompt medical consultation. This underscores the role of physical discomfort in driving healthcare utilization, independent of the health system or external factors such as pandemics. Osteoporosis, often undetected until a fracture occurs, can lead to severe pain, drastically affecting quality of life, mobility, and independence^33,34^.

Restricted mobility, limited access to therapeutic services, and heightened stress during the lockdown may have exacerbated osteoporotic symptoms. Additionally, virus exposure fears might have led patients to postpone seeking treatment, aggravating their condition^35,36^. Binary logistic regression modeling revealed that patients experiencing pain were significantly more likely to seek care once restrictions were lifted, highlighting the critical role of pain management in osteoporosis treatment and the need for innovative strategies to ensure continued care, such as telemedicine and digital pain management programs^37^.

The link between symptom severity and health-seeking behavior is well-documented^38,39^. Our analysis revealed that with each decrease in symptom severity, the likelihood of seeking care post-lockdown decreased. This suggests that patients with severe symptoms prioritized immediate care, whereas those with milder symptoms might have delayed treatment due to perceived non-urgency or a belief in self-management. Healthcare systems should therefore ensure accessible care for patients with severe symptoms, even during disruptions like a pandemic. Telemedicine and remote management programs can be invaluable in managing symptoms when in-person consultations are challenging.

Anxiety emerged as a significant factor driving medical consultations in our study, where heightened health-related anxiety, stemming from limited healthcare access, virus contraction fears, and the psychological impacts of isolation, increased the likelihood of seeking outpatient care post-lockdown by over five times. This aligns with literature indicating that anxiety can lead to increased healthcare utilization, often for reassurance or symptom management concerns^40^. These findings underscore the need for integrated mental health support within healthcare services, particularly for managing chronic conditions like osteoporosis during healthcare disruptions.

Though not significantly different between two groups, patients' reasons for their first post-lockdown clinic visit, predominantly 'bad or aggravating symptoms,' seem to echo the binary logistic regression results, emphasizing symptom severity's role in healthcare-seeking behaviors. Other top reasons, such as prolonged absence of follow-up consultations and medication needs, suggest that continuity of care is a critical concern, potentially heightened by anxiety. The explicit mention of 'feeling anxious' as a reason for clinic visits further highlights the necessity of addressing emotional well-being in patient care.

Interestingly, the reasons for seeking post-lockdown osteoporosis care, notably prolonged absence from follow-up consultations (48.1%) and the need for medication prescriptions (47.6%), underscore the critical role of consistent healthcare engagement. This trend may indirectly reflect heightened anxiety levels, prompting patients to proactively manage their condition through follow-up visits and medication management. Furthermore, the substantial percentage of patients citing 'feeling anxious' (42.6%) as a reason for their visit directly links anxiety with increased healthcare utilization, emphasizing the need to address emotional well-being alongside physical health during healthcare disruptions. The findings on 'rehabilitative therapy' (42.1%) and 'follow-up visits' (40.6%) suggest patients' eagerness to regain control over their health post-lockdown. These motivations, while not directly captured in the regression model, highlight the broader spectrum of patient needs and concerns that extend beyond immediate symptom management.

It is worth noting that the study indicates that physical activity levels did not significantly influence the decision to seek care post-lockdown, despite overall reduced activity among patients as per the IPAQ results. This could suggest that the immediate perceived need for medical attention may not be directly linked to changes in physical activity, or that other factors such as pain, symptom severity, and anxiety may have a more pronounced impact on care-seeking behaviors. The reliance on self-reported data for physical activity assessment, collected months after the lockdown, introduces the potential for recall bias, which could affect the accuracy of these findings.

The sudden surge in osteoporosis outpatient visits prompted our clinic to maximize its service capacity for meeting the increased demand, including extending clinic hours, deploying additional healthcare providers, and enhancing telehealth services. This awakening experience caused the hospital administrators to realize certain essential needs in such disaster events, for example flexibility and adaptability in healthcare delivery and ability to rapidly scale up service capacity and implement alternative modes of care delivery, which may significantly enhance a healthcare system's preparedness for similar scenarios in the future.

### Limitations

There are several limitations to our study. Notably, the initial method used for sample size estimation was more appropriate for prevalence studies rather than for investigating associations between binary outcomes and predictor variables. This methodological oversight was identified during the peer review process, leading us to reassess our sample size using a more suitable approach as described by Tushar Vijay Sakpal et al. Though our reassessment confirmed that our actual sample size was more than sufficient to detect large and medium (0.5) effect sizes, it was not adequately powered to reliably detect small (0.2) effect sizes. This limitation is particularly relevant when considering the nuanced factors that may influence a patient's decision to seek care, as smaller effect sizes might represent subtle yet clinically meaningful associations. As a result, while our findings offer significant insights into the primary factors influencing clinic visits during the post-lockdown period, they should be interpreted with caution, especially regarding associations or differences of a smaller magnitude. Also, the external validation relied on qualitative feedback from a small group of osteoporosis specialists, which may not accurately represent the broader situation. Their responses were based on their personal observations, which might be influenced by biases or individual perspectives. Additionally, the sample size of seven specialists, while providing an initial indication, is not statistically significant and may not capture the full variability across the country. The internal validation and subsequent survey using the data of a single clinic could limit the generalizability of our findings. Furthermore, self-reported data used in our analyses are inherently subject to recall bias and subjective interpretation, especially given that the survey was months after the lockdown. Therefore, future research should consider a larger, multi-center study to better capture the variation in outpatient volume across different settings and further confirm our findings.

## Conclusions

The unexpected surge in osteoporosis outpatient visits after COVID-19 lockdown suggests vast latent demand for osteoporosis care accumulated during the pandemic. Four significant factors predict post-lockdown outpatient care-seeking, including being female, experiencing pain and aggravating symptoms, and heightened anxiety levels. Even though physical activity level dropped during lockdown, it fails to predict care-seeking. The findings underscore the resilience of osteoporosis patients to resume regular care despite disruption and stress the substantial backlog of unmet healthcare needs. The study provides insights for effective management and anticipation of similar challenges in future public health emergencies.

## Data Availability

The raw data used in the study are available on reasonable request to the corresponding author.

## Appendix 1

See Table 6.

**Table 6:** Demographics and responses of osteoporosis specialists surveyed on external validation (N = 7)

#	Gender	Age	City, region	Response^*^
1	F	30–45	Chengdu, West China	1
2	F	30–45	Shanghai, East China	1
3	M	30–45	Shanghai, East China	1
4	F	45–60	Guangzhou, South China	2
5	M	45–60	Shenzhen, South China	2
6	M	30–45	Shenyang, North China	1
7	F	30–45	Urumuqi, West China	1

*1, significant increases in osteoporosis outpatients after COVID-19 lockdown; 2, some increases in osteoporosis outpatients after COVID-19 lockdown.

## Appendix 2

See Table 7.

**Table 7:** Variable assignments of logistic regression model

Variable	Assignment	Variable	Assignment
Visit to osteoporosis clinic after COVID-19 lockdown		Osteoporosis-related symptoms during COVID-19 lockdown	
No	0	None	1
Yes	1	Fracture	2
Age, yr		Pain	3
< 30	1	Tooth loosening	4
30–45	2	Reduced body weight	5
45–60	3	Ear ringing	6
> 60	4	Symptom change than before COVID-19 lockdown	
Gender		Much worse	1
Male	1	Somewhat worse	2
Female	2	Not much change	3
BMI		Somewhat improved	4
< 18.5	1	Much improved	5
18.5-24.9	2	Experience of anxiety during COVID-19 lockdown	
> 24.9	3	Not at all	1
Education level		A little	2
Illiterate	1	Very much	3
Primary school	2	Experience of depression during COVID-19 lockdown	
High school	3	Not at all	1
College or over	4	A little	2
Marital status		Very much	3
Single	1	Mental state change than before COVID-19 lockdown	
Married	2	Much worse	1
Divorced	3	Somewhat worse	2
Widowed	4	Not much change	3
Residency		Somewhat improved	4
Urban	1	Much improved	5
Rural	2	Use of osteoporosis-related medical services during COVID-19 lockdown	
Living condition		None	1
Alone	1	Online consultation	2
With spouse/offspring	2	Medication home delivery	3
Insurance		In-person outpatient consultation	4
None	1	Hospitalization	5
Urban resident/employee insurance (social, medical insurance)	2	Home visit care	6
Rural cooperative medical insurance	3	Physical activity during COVID-19 lockdown	
Commercial insurance	4	Low	1
Time of osteoporosis, yr		Moderate	2
< 1	1	High	3
1–3	2		
> 3	3		
